# Gender-focused analysis and opportunities for upgrading within Vietnam's smallholder pig value chains

**DOI:** 10.3389/fvets.2022.906915

**Published:** 2022-08-09

**Authors:** Nga Nguyen-Thi-Duong, Hung Pham-Van, Ha Duong-Nam, Huyen Nguyen-Thi-Thu, Trung Ninh-Xuan, Sinh Dang-Xuan, Fred Unger, Hung Nguyen-Viet, Delia Grace

**Affiliations:** ^1^Faculty of Economics and Rural Development, Vietnam National University of Agriculture, Hanoi, Vietnam; ^2^Animal and Human Health Program, International Livestock Research Institute, Hanoi, Vietnam; ^3^Natural Resources Institute, University of Greenwich, Kent, United Kingdom

**Keywords:** gender, food safety, value chain, swine, Vietnam

## Abstract

Gender influences participation in food value chains (VCs) with implications for VC upgrading. This study investigated roles as well as differences in production activities, awareness, training, and attitudes between men and women in Vietnam's smallholder pig VCs. Data were gathered from a survey of 1,014 actors in different nodes along the chain, and the results showed that both men and women participated in all nodes of the VCs. Women were mainly in charge of routine husbandry activities (e.g., preparing feed, feeding animals, and cleaning pig pens) and participated in input supply (34.7%), pig production (60.2%), pork processing (63.6%), retailing (93.1%), and home preparation and cooking (100%). Men were more often responsible for tasks requiring strength, knowledge, and skills (e.g., disease management) and had greater involvement in larger-scale farming (60–80%) and slaughtering activities (98.0%). Selling of pigs was handled by both genders, but mainly men (73–80%), especially in larger farms. Likely challenges for upgrading pig VCs include limited training for producers, low concern for occupational health risks in all nodes, and misperceptions about food safety. In general, this study found no clear evidence of perceived gender inequality in the smallholder pig VCs in lowland Vietnam. Gendered upgrading in pig VCs should focus on improving women's ability to access veterinary services and animal disease management and on educating relevant VC actors about occupational health risks.

## Introduction

In recent years, globalization and trade liberalization have led to more complex food systems in developing countries, accompanied by rising penetration of the modern retail sector. This offers the opportunity for those involved in domestic food production to expand and integrate with the emerging commercial sector, thereby increasing (and potentially stabilizing) their income and also creating more opportunities for rural employment, especially for women, including upgrading production (1). In this paper, value chain (VC) upgrading in small-scale pig value chains, refers to improvement of VC performance and outputs, by optimizing and balancing involvement of men and women, equipping men and women with better skills, knowledge, practices, and supporting enabling policies, investments and culture. That would also enable small-scale actors to retain existing income and generate more, while supporting other societal objectives such as health, sustainability, animal welfare and gender equity.

According to Vietnam's Ministry of Agriculture and Rural Development, pig production in Vietnam accounts for 71% of the livestock sector, providing livelihoods for around 2.4 million small-scale pig-raising households (2). Until recently, these smallholdings produced about 80% of the total market supply of pork (3), which was predominantly sold through the traditional market (4). However, in the last few years, Vietnam's smallholder pig VC has faced increasing competition from the growing number of large-scale producers in the country, led by large vertically-integrated contractors (5). Another challenge has been the incursion of African swine fever outbreaks, a highly contagious and lethal pig disease, since 2019 (6). Disease outbreaks can stimulate farm modenisation and intensification, but African swine fever is unlikely to lead to the complete replacement of small-scale producers with modern commercial farms (7). The income and opportunities available to smallholder pig producers will therefore continue to be of vital importance for local stability and development.

Upgrading of VCs is an important step forward for rural communities, but it requires various players within the chain to overcome significant obstacles. Among these obstacles are traditional gendered roles. Women are major contributors within livestock VCs, but they face numerous constraints that limit their ability to achieve optimal livestock production and agricultural development (8). A growing body of evidence suggests that VC upgrading strategies that fail to take gendered participation levels into account under-achieve (9). Therefore, understanding gender issues in the traditional food system is critical for developing successful upgrading strategies. While this issue has been studied in horticulture (10, 11), little research has been conducted on women's roles in livestock farming (12) and their impact on VC upgrading in developing countries. This study was conducted to investigate gendered roles and differences in production activities, awareness, training, and attitudes between men and women in the smallholder pig VC in Vietnam. By leveraging both this knowledge and examples from the literature, we then present several possible strategies for gender-aware and informed upgrading of the smallholder pig production chain in Vietnam and in similar contexts worldwide, with a parallel focus on incorporating improved food safety.

## Materials and methods

### Study design and location

This cross-sectional study was conducted between 2013 and 2014. Two provinces in northern Vietnam were selected to reflect differences in Vietnam's small-scale pork production spectrum, with Nghe An being more traditional and Hung Yen being more emerging and urban in orientation. The Nghe An province is situated in the North Central Region of Vietnam, while Hung Yen is located on the outskirts of Hanoi (the capital city of Vietnam). Within both provinces, three districts were selected based on their representation of different degrees of integration among rural, peri-urban and urban pig VCs (Supplementary Figure 1). Finally, among the communes with the highest pig densities, three communes in each district were selected.

### Target population and sample sizes

This study targeted the key actors along the smallholder pork production chain, including (i) input suppliers, who sold feed, veterinary medicines and other farm equipment for pig producers through shops in the village or commune; (ii) pig producers, used either farrow-to-finishing or fattening system to produce finished pigs for sale; (iii) slaughterhouse owners, who often bought live pigs from producers, and managed slaughtering pigs to sell pig carcass to retailers, processor, etc.; (iv) pork processors, who processed different pork products, such as meat loaf, pâte, sausage; (v) pork retailers, who mainly sold pork to consumers buying from traditional markets, and (vi) pork consumers, referred to regular consumers who often eat pork and also in charge of buying, preparing and cooking pork at home.

The sample sizes were calculated based on a two proportion comparison. Among pig producers, we assumed that the difference in proportions of male and female involvement in various production activities was 15%. Confidence level of 95% and power of test of 80% were used. By considering 5% non-response rate, the final sample sizes for pig producers and pork consumers were 420 and 416, respectively. Pig producers and consumers were randomly selected from a household list that was provided by local authorities. The participants for the other groups were recruited based on suggestion of local veterinary staff and the availability of actors in the 18 selected communes. In total, 31 input (feed, piglets, and veterinary drugs) suppliers, 51 slaughterhouse owners, 22 pork processors, and 74 pork retailers participated in the survey. During the survey, one participant may have several roles in the VC, such as slaughterhouse owners could also raise pigs, process, or sell pork at market. However, in selecting the respondents, we applied the rule that enumerator pre-defined and selected she/he who performed the most important function in VC. For instance, if she/he engaged in pig raising, but the main time and task of her or him was to sell pork at the market, so she/he belonged to pork retailer group, not pig producer group.

### Questionnaires and interviews

Semi-structured questionnaire forms were developed for each group of VC actors by the research team. Then, to evaluate and finalize, these questionnaires were pre-tested in Gia Lam (a peri-urban district of Hanoi), where the pig VC context is similar to the districts included in the study. The questionnaires used were under a larger survey of a food safety project, called PigRISK (13), and the questionnaires could be provided upon request. The main parts of the questionnaire consisted of (i) general demographic information, e.g., age, gender, and education; (ii) actors' activities and business operations (inputs and outputs), e.g., number of pigs or volume of pork sold; (iii) knowledge, attitudes, and practices about food safety and health risk; and (iv) constraints, opportunities, and recommendations toward the upgrading of VCs. Trained enumerators conducted in-depth face-to-face interviews in Vietnamese, lasting one to 2 h, with the VC actors. Written informed consent was obtained from all individual participants before starting the interviews.

### Data management and analysis

Data collected from interviews were managed in Microsoft Excel (2010) spreadsheets. The pig production scale was defined as small, medium, and large, with the number of pigs raised in the latest cycle being ≤ 10, 11–30, and > 30, respectively. Descriptive and comparative statistics were employed with *t*-tests and chi-square tests where appropriate, using a significance level of *p* ≤ 0.05. Ethical approval for this study was obtained from the Institutional Review Board of the Hanoi School of Public Health (No.148/2012/YTCC-HD3).

## Results

### Characteristics of respondents and smallholder pig value chains in Vietnam

The average ages of the interviewed VC actors ranged from 46 to 51 years old for men, and 39 to 51 years old for women. Male VC actors tended to be older than their female counterparts, except for the retailer and consumer groups. While the proportions of male and female respondents were almost equal in the input supplier, pig producer, slaughterhouse owner, and pork processor groups, female respondents accounted for 93.2 and 80.1%, respectively, of the retailer and consumer groups. The majority of producers, slaughterhouse owners, processors, retailers, and consumers had completed secondary and high school. Female respondents in these groups had less education than male. For example, the proportions of female respondents having either no education or having completed primary school only were 8, 2.9, and 12% for slaughterhouse owners, retailers, and consumers, respectively, while these figures were 0, 0, and 4.5% for male respondents. VC actors had been in business from 6.8 to12 years. The gender ratio of those most responsible for labor varied among groups—women were minimally involved in slaughterhouses, for instance, but very highly involved in pork retailing at markets and food handling at home (Table 1). The smallholder pig value chain in Vietnam covers several main functions which are operated by different actors, including input supply, production, collection, slaughtering, processing, and retailing, which is illustrated in Supplementary Figure 2.

**Table 1:** Demographic characteristics of survey respondents.

**Characteristics**	**Input supplier (*n* = 31)**	**Pig producer (*n* = 420)**	**Slaughterhouse owner** **(*n* = 51)**	**Pork processor (*n* = 22)**	**Pork retailer** **(*n* = 74)**	**Pork consumer (*n* = 416)**
Average age *(years old)*						
Female respondents	46.5	48.2	49.3	50.6	46.4	48.0
Male respondents	38.8	45.7	44.7	46.4	47.2	50.7
Female respondents *(%)*	54.8	51.4	49.0	63.6	93.2	80.1
Education (Male/Female, %)						
Illiteracy, primary school	0/0	4.9/2.8	0/8.0	0.0	0/2.9	4.5/12.0
Secondary, high school	42.9/29.4	88.2/91.7	100/92.0	100/100	100/97.1	72.4/67.5
Vocational level	28.6/58.8	2.9/3.7	0/0	0/0	0/0	8.1/7.2
College, university	28.6/11.8	3.9/1.9	0/0	0/0	0/0	15.0/13.3
Proportion participating in other value chain roles *(%)*						
Input supplier	100	7.4	0.0	0.0	0.0	0.0
Pig producer	54.8	100	58.8	13.6	27.0	28.1
Pig collector	3.2	1.2	5.9	0.0	1.4	0.0
Slaughterhouse owner	0.0	1.7	100	22.7	40.5	0.0
Processor	0.0	0.0	23.5	100	0.0	0.0
Retailer	0.0	1.4	96.1	31.8	100	1.9
Years of doing business *(years)*	10.6	9.9	12.2	6.8	12.0	na
Physical labor responsibility (male/female, %)	65.3/34.7	39.8/60.2	98.0/2.0	36.4/63.6	6.8/93.2	na

### Gendered participation at different steps of the pig production value chain

#### Input suppliers

Men more commonly owned the registration of veterinary drug shops (78.6%) but Hung Yen had a lower men-owned shop registration than Nghe An (71.4 and 85.7%, respectively). However, women were more often responsible for managing these shops (71.4%) In the feed supplier group, shop registration and management were more evenly split between men and women. The same trend was applied for both provinces (Table 2).

**Table 2:** Gendered participation levels in different activities by provinces and actors along pig value chains.

**Value chain actors and involved activities**	**Hung Yen**			**Nghe An**			**Overall**			
	**Male**	**Female**	Both male and female***	**Male**	**Female**	**Both male and female**	**Male**	**Female**	**Both male and female**	*p*-value*
**Animal feed suppliers (*****n*** **=** **17)**	***n*** **=** **6**	***n*** **=** **2**		***n*** **=** **4**	***N*** **=** **5**		***n*** **=** **7**	**n** **=** **10**		
Own the shop registration (%)	62.5	37.5	0	44.4	55.6	0	52.9	47.1	0	1.000
Main person managing the shop (%)	62.5	37.5	0	55.6	44.4	0	58.9	41.1	0	0.637
**Veterinary drug suppliers (*****n*** **=** **14)**	***n*** **=** **2**	***n*** **=** **5**		***n*** **=** **2**	***n*** **=** **5**		***n*** **=** **7**	**n** **=** **7**		
Own the shop registration (%)	71.4	28.6	0	85.7	14.3	0	78.6	21.4	0	0.132
Main person managing the shop (%)	28.6	71.4	0	28.6	71.4	0	28.6	71.4	0	0.286
**Pig producers (*****n*** **=** **420)**	***n*** **=** **135**	***n*** **=** **77**		***n*** **=** **69**	***n*** **=** **139**		***n*** **=** **204**	**n** **=** **216**		
Can access credit provided by banks if needed (%)	88.1	83.1	na	76.8	69.1	na	84.3	74.1	na	0.014
Currently borrowing credit from bank (%)	34.8	37.7	na	29.0	23.7	na	32.8	28.7	na	0.416
Can loan a total amount of money (million VND**)	42.3	50.6	na	56.1	24.6	na	46.5	36.7	na	0.999
Attended training in the past year (%)	30.4	24.7	na	17.4	7.2	na	26.0	13.4	na	0.002
Number of trainings attended (times/year)	0.8	0.7	na	0.7	0.6	na	0.8	0.6	na	0.009
Production cost (US$/100 kg of live pigs)	155.6	151.4	na	148.2	146.9	na	153.1	148.5	na	0.624
Selling price (US$/100 kg of live pigs)	194.1	190.4	na	181.9	183.3	na	190.0	185.8	na	0.319
Total income (US$/production cycle)	534.6	264.8	na	250.4	67.5	na	438.5	137.8	na	<0.001
**Slaughterhouse owners (*****n*** **=** **51)**		***n*** **=** **23**			***n*** **=** **28**	***n*** **=** **26**	**n** **=** **25**		
Communicate with farmers	73.9	4.3	21.7	67.9	3.6	28.6	70.6	3.9	25.5	<0.001
Buy and transport pigs	87.0	0.0	13.0	78.6	0.0	21.4	82.4	0.0	17.6	<0.001
Care for pigs in lairage	0.0	34.8	65.2	10.7	42.9	46.4	5.9	39.2	54.9	0.009
Clean tools/floor	0.0	78.3	21.7	0.0	64.3	35.7	0.0	70.6	29.4	<0.001
Slaughter pigs	8.7	0.0	91.3	0.0	0.0	100.0	3.9	0.0	96.1	1.000
**Pork processors (*****n*** **=** **22)**		***n*** **=** **11**			***n*** **=** **11**	***n*** **=** **8**	**n** **=** **14**		
Buy and transport pork	18.2	18.2	63.6	9.1	36.4	54.5	13.6	27.3	59.1	0.191
Prepare and process pork	18.2	9.1	72.7	0.0	9.1	90.9	9.1	9.1	81.8	1.000
Clean tools/equipment	0.0	9.1	90.9	9.1	36.4	54.5	4.5	22.7	72.8	0.110
Sell products	0.0	9.1	90.9	0.0	36.4	63.6	0.0	22.7	77.3	0.110
**Pork retailers (*****n*** **=** **74)**	***n*** **=** **5**	***n*** **=** **29**		***n*** **=** **0**	***n*** **=** **40**		***n*** **=** **5**	**n** **=** **69**		
Buy and transport pork	5.9	38.2	55.9	0.0	30.0	70.0	2.7	33.8	63.5	0.316
Clean tools/equipment at the shop	2.9	97.1	0.0	0.0	85.0	15.0	1.4	90.5	8.1	<0.001
Sell pork	5.9	88.2	5.9	0.0	82.5	17.5	2.7	85.1	12.2	<0.001
**Pork consumers (*****n*** **=** **416)**		***n*** **=** **208**			***n*** **=** **208**	***n*** **=** **83**	**n** **=** **333**		
Decide to buy pork	12.0	85.6	2.4	17.8	80.8	1.4	14.9	83.2	1.9	<0.001
Buy and transport pork from market	5.8	79.3	14.9	9.6	73.6	16.8	7.7	76.4	15.9	<0.001
Prepare and process/cook pork	10.1	80.3	9.6	10.6	71.2	18.3	10.4	75.7	13.9	<0.001

Na, not applicable; VND, Vietnamese dong; (*) p-values were derived from comparison between male and female groups, using t-tests, Fisher's exact tests, or chi-square tests where appropriate; (**) exchange rate, US $1 = 20,400 VND (at the time of the survey); (***) referred to the joint and equal decision, management or involvement of activities.

#### Pig producers

Ownership of production equipment, assets, and livestock (including pigs) was considered to be jointly owned by male and female (often husband and wife), and the income generated from livestock production was similarly shared in the household. In borrowing capital, nearly 80% of farm households could access credit provided by banks, and approximately 30% of those surveyed were borrowing from banks. There was no significant difference in loan amounts between male and female respondents. Related to attending training on pig production in the past year, proportion of male respondents (26.0%) was significantly higher than that of female respondents (13.4%). Pig producers in Hung Yen attended more trainings in the past year compared to Nghe An (Table 2).

#### Slaughterhouse owners

Slaughtering units in rural areas were usually operated by married couples. Men typically led most activities, including communicating with farmers to buying and transporting pigs from farm to place of slaughter (70.6% and 82.4%, respectively). Women supported men in all of these tasks, especially in feeding pigs in lairage, preparing equipment (e.g., knives, containers, and hot water), and cleaning slaughtering tools and floors. Both males and females were involved in slaughtering pigs—men largely handled bleeding, flaying, and splitting the carcass, while women supported men in eviscerating and splitting (96.1%), and those figures were generally lower in Hung Yen as compared to Nghe An (Table 2).

#### Pork processors

The main processed products from pork are meat loaf, fermented pork, sausages, dry meat, pâte, and cooked organs (mainly intestines). Meat processors were often scarce in the study areas (i.e., 1–2 pork processors in each surveyed commune). Men and women usually worked together in preparing and processing pork (81.8% of processing units) and this figure was higher in Hung Yen as compared to Nghe An. Women were mainly involved in pork processing tasks, especially procuring, and cooking pork and retailing products (22.7 to 27.3%, Table 2).

#### Pork retailers

Most pork retailers were women (92.3%, Table 1) who worked either as exclusive retailers or as slaughterer-cum-retailers. However, men participated more in selling pork in Hung Yen than in Nghe An. The role of slaughterer-cum-retailer was quite common in both rural and urban traditional markets in Vietnam. Buying and transporting pork from slaughterhouses was a task that could be shared by males and females (63.5%). After this step, most of the subsequent tasks of cleaning the pork shop, selling pork, and sometimes shipping pork to consumers were carried out by the female retailers (85.1–90.5%, Table 2).

#### Consumers

Seventy-five percent of pork buyers were females, and they were also responsible for processing and cooking at home. The decision to buy pork for household consumption was usually made by females (83.2%), and less often by males (14.9%). The tasks of buying and transporting pork from market or preparing, processing, and cooking pork at home were sometimes shared by males and females in the family (15.9 and 13.9%, respectively, Table 2).

### Gendered participation in deciding and performing tasks in pig production

**Figure 1:**
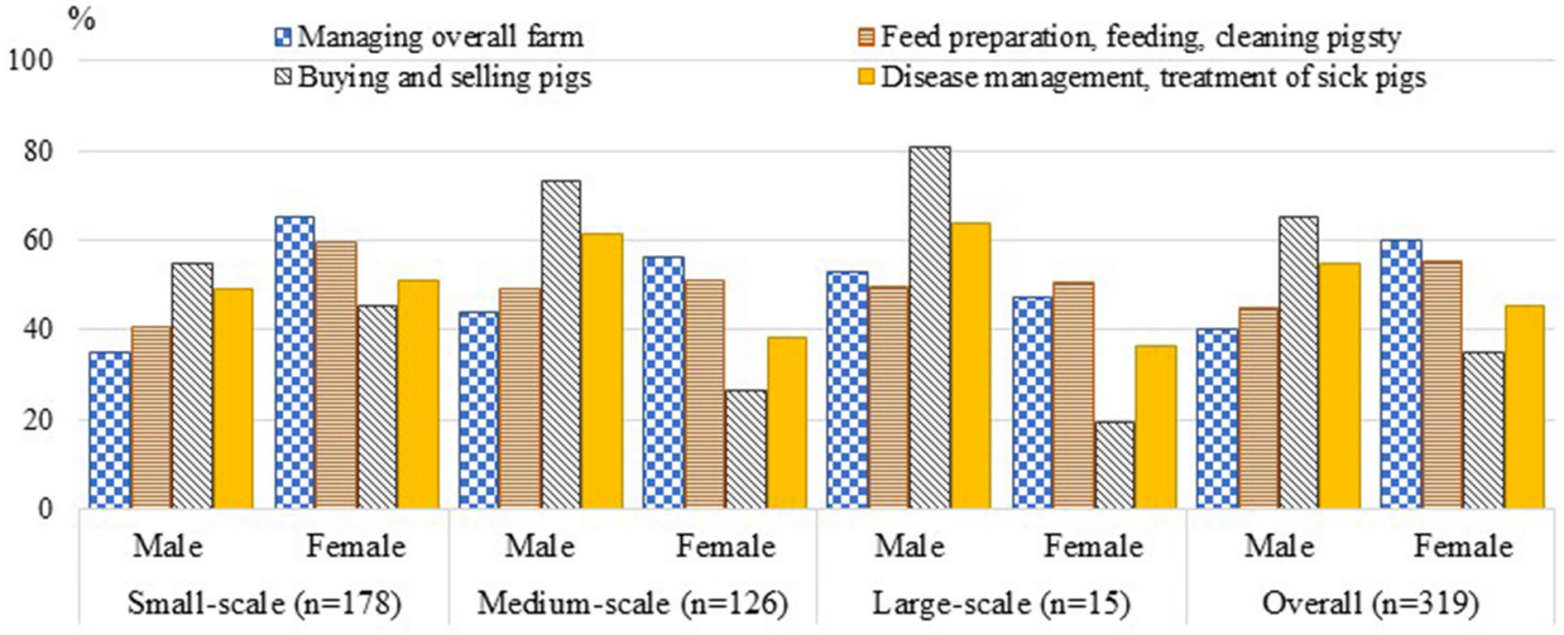
Gendered participation in deciding and performing tasks in pig farming (% time allocated). Small-, medium-, and large-scale farms were defined as having respectively ≤ 10, 11–30, and > 30 pigs in the latest cycle.

Comparing the participation of males and females across different production scales, males had a higher involvement in larger operations compared to their female counterparts, who were mainly in charge of small-scale farms. Both men and women were involved in all pig production activities, but women were more often responsible for routine, daily husbandry activities (feed preparation, feeding, and cleaning pigsties), and men were more responsible for non-routine and less frequent husbandry activities such as disease management and selling pigs. Meanwhile, in larger-scale farms, treating sick animals (detecting disease, prescribing and buying drugs, and administering drugs to pigs) and vaccinating pigs were mainly conducted by males (61–64%), while in small-scale farms, females could be equally responsible for these tasks. Similarly, the decision to sell pigs was normally reached jointly by both men and women on small-scale farms—conversely, in most large-scale farms, males made the decision to buy or sell pigs by themselves (73–80%, Figure 1).

Regarding the management of disease risk in pig production, a significantly higher percentage (11.4%) of women-owned pig farms reported buying piglets that they later realized were sick, compared to their male-owned counterparts (5.8%). However, male-owned pig farms reported a significantly higher percentage of sick piglets (32.2%) compared to female-owned ones (23.6%). Between male- and female-owned pig farms, the percentages of sick growing pigs were nearly equal (5.7% and 4.4%, respectively), as were the numbers of dead pigs (1.9 and 1.8 pigs/farm/year, respectively, Supplementary Table 1). The average monetary loss due to sick and dead pigs in the previous year was reported as US $ 92, and this amount was not significantly different between male- and female-owned farms. However, as the production scale of female-owned farms is comparatively smaller, it could be inferred that women bear higher disease risks in pig production.

### Gendered knowledge and perceptions of food safety and occupational health risks

Almost two-thirds of producers had heard about food safety requirements in pig production. However, only one-third of them (both men and women) agreed that pig diseases could be transmitted to humans, while 32.3% did not agree and 35.4% did not know. Thirty-six percent of pig producers also perceived that eating pork from diseased pigs could cause human illness; the rest either did not know or reported that eating pork from disease pigs does not cause harm. This likely impacted farmers' behavior in the treatment of dead pigs (usually dead from disease). Both males and females perceived burying of dead pigs as the most practical treatment method (73.5 and 80.6%, respectively), while males were more likely to perceive burning as a practical method (11.3 and 5.6%, respectively). Finally, both men and women infrequently consulted veterinarians (3.4 and 4.2%, respectively), and female respondents were significantly less likely to sell dead pigs to slaughterhouses (5.6 and 13.2%, respectively, Table 3).

**Table 3:** Knowledge and perceptions of value chain actors on food safety and zoonotic diseases, by gender (%).

**Knowledge and perceptions**	**Hung Yen**		**Nghe An**		**Both provinces**			
	**Male**	**Female**	**Male**	**Female**	**Male**	**Female**	**Overall**	***p*-value^†^**
**Pig producers**	***n*** **=** **135**	***n*** **=** **77**	***n*** **=** **69**	***n*** **=** **139**	***n*** **=** **204**	***n*** **=** **216**	***n*** **=** **420**	
Heard about food safety requirements in pig production (Yes)	75.4	64.9	68.1	68.3	73.7	67.7	69.9	0.220
Pig diseases can be transmitted to humans	
Yes	29.9	19.5	39.7	38.1	28.8	34.4	32.3	0.285
No	30.6	41.6	30.9	29.5	33.3	31.7	32.3	0.842
Don't know	39.6	39.0	29.4	32.4	37.8	34.0	35.4	0.458
Eating pork from diseased pigs could cause illness	
Yes	42.2	18.2	39.1	38.8	38.5	34.8	36.2	0.454
No	31.9	54.5	55.1	58.3	44.9	50.8	48.6	0.231
Don't know	25.9	27.3	5.8	2.9	16.7	14.4	15.2	0.603
Dead pigs should be (*multiple selection*)	
Used for own consumption	0.7	2.6	2.9	1.4	1.5	1.9	1.7	1.000
Thrown away	7.4	9.1	4.3	4.3	6.4	6.0	6.2	1.000
Burned	10.4	5.2	13.0	5.8	11.3	5.6	8.3	0.052*
Buried	74.8	66.2	71.0	88.5	73.5	80.6	77.1	0.110
Disposed of according to veterinarian advice	0.7	0.0	8.7	6.5	3.4	4.2	3.8	0.890
Sold to a slaughterhouse at a cheaper price	20.0	7.8	0.0	4.3	13.2	5.6	9.3	0.011**
Given to neighbors	5.2	9.1	11.6	2.2	7.4	4.6	6.0	0.331
**Slaughterhouse owners** (Yes)	***n*** **=** **12**	***n*** **=** **11**	***n*** **=** **14**	***n*** **=** **14**	***n*** **=** **26**	***n*** **=** **25**	***n*** **=** **51**	
Heard about food safety requirements in slaughtering pigs	16.7	18.2	14.3	28.6	15.4	24.0	19.6	0.499
Presence of dirt on cloth or tools does not cause harm	91.7	100	78.6	85.7	84.6	92.0	88.2	0.668
It is easy to keep slaughterhouse, carcasses, and equipment clean	75.0	90.9	71.4	100	73.1	96.0	84.3	0.050**
Slaughtering could have negative impacts on the environment	54.5	45.5	50.0	50.0	50.0	48.0	49.0	1.000
If meat is well-cooked, then it is always safe to eat	91.7	90.9	92.9	85.7	92.3	88.0	90.2	0.668
Pig diseases can be transmitted to humans	66.7	72.7	78.6	71.4	73.1	72.0	72.6	1.000
People performing slaughtering jobs are more likely than others to get sick	27.3	18.2	28.6	35.7	26.9	28.0	27.5	1.000
**Pork processors** (Yes)	***n*** **=** **6**	***n*** **=** **5**	***n*** **=** **2**	***n*** **=** **9**	***n*** **=** **8**	***n*** **=** **14**	***n*** **=** **22**	
Pork for processing should be from healthy pigs	100	100	100	100	100	100	100	1.000
Safe products are free of food additives	16.7	80.0	50.0	55.6	25.0	64.3	50.0	0.183
Short storage time is safer for pork products	83.3	80.0	100	77.8	87.5	78.6	81.8	1.000
Pork can be contaminated with bacteria during transportation	0.0	0.0	0.0	11.1	0.0	7.1	4.5	1.000
Pork can be contaminated with bacteria during selling at the market	0.0	20.0	0.0	22.2	0.0	21.4	13.6	0.273
**Pork retailers** (Yes)	***n*** **=** **5**	***n*** **=** **29**	***n*** **=** **0**	***n*** **=** **40**	***n*** **=** **5**	***n*** **=** **69**	***n*** **=** **74**	
Presence of dirt on cloth or tools does not cause harm	60.0	59.0	0.0	53.0	60.0	55.1	55.4	1.000
It is easy to keep shop, pork, and equipment clean	100	59.0	0.0	60.0	100	59.4	62.2	0.150
If pork is labeled by an inspector, then it is always safe to eat	0.0	66	0.0	75.0.	0.0	70.6	65.8	0.003**
If pork is well-cooked, then it is always safe to eat	0.0	72.0	0.0	73.0	0.0	72.5	67.6	0.003**
People selling pork are more likely than others to get sick	0.0	21.0	0.0	20.0	0.0	20.3	18.9	0.576

(†)p-values were derived from comparison between male and female groups, using Fisher's exact tests, or chi-square tests where appropriate; (*) p < 0.1; (**) p < 0.05.

Few slaughterhouse owners (19.6%) had heard about food safety requirements in pig slaughtering, and they lacked awareness of certain risk factors for contamination (such as the use of dirty cloth or tools during slaughtering). Almost all female respondents (96.0%) and most male respondents (73.0%) agreed that it is easy to keep the slaughtering process clean. Interestingly, two-thirds of all respondents agreed that “Pig diseases can be transmitted to humans”, but only 27.5% of them agreed that slaughtering pigs carries an occupational disease risk (Table 3).

All processors of both genders agreed that food safety requires that raw meat must come from healthy pigs. Female processors were more likely to agree that safe products are free of food additives (64.3%), compared to 25.0% of male processors. Meanwhile, a majority of pork retailers (62.3%) reported that it was easy to keep their shop, pork, and equipment clean; however, 55.4% of them still believed that the presence of dirt on cloth or tools could not cause harm. A significantly higher proportion of female retailers (72.5%) agreed that pork that is well-cooked or labeled by an inspector is always safe to eat. Finally, few retailers (18.9%) reported that there were health risks involved in handling raw meat daily (Table 3).

## Discussion

These findings show that women perform the bulk of activities in the smallholder pig value chain (VC) in Vietnam, with major roles in production, processing, retailing meat, and processing food at home. We found, women dominated sale, although others report whoever is available at an input sells drugs, whether man or woman (14). Farmers consult drug sellers on treating sick animals, so, training for the women who run the shops is important. Moreover, drug sellers could be incorporated into surveillance if they had capacity and incentives to report on treatment seeking. Pig slaughtering is considered physically hard work and is therefore done mostly by men; similar results are found in most livestock VCs, including pig (1, 15, 16). Though men and women work jointly in the retailing node, this study found, as did Grace et al., that women are the main laborers (1, 15, 16). Therefore, upgrading of smallholder pig VCs is important for women in terms of jobs and income.

Joint ownership of pigs and production equipment between males and females (often husbands and wives in the household) is common in rural Vietnam. This differs from other countries such as Uganda, where pigs are typically owned by women rather than men (17), and Nicaragua, where more than 50% of women owned pigs (18). According to a study by the Institute for Social Development Studies (19), ownership or co-ownership of a family's most valuable property plays an important role in bargaining power relations between spouses. However, this is not the case for livestock (including pigs) in some African countries, where men typically control sale decisions (8).

Women are known to play a major role in pig production (16, 20, 21). In countries with free-range pigs, like New Guinea and Uganda, herding pigs falls mainly to women (22, 23). Women contribute more significantly to routine, daily activities including food preparation, feeding, and cleaning pigsties (14, 17, 24), and these are considered to be exclusively tasks for women (25). They align with women's caretaking roles within the house and are not physically demanding. Men are more involved in activities that are physically demanding, require specialist skills and knowledge, and occur outside the homestead (17, 20, 24, 25). Other studies found that, despite women's involvement in pig production, men receive more support (primarily training) from agricultural extension services, (16, 17, 26, 27). Thus, improving women's ability to access all services and providers is essential for upgrading VCs.

Literature shows that male share of labor increases with production scale (14, 16, 28), particularly in production systems with high value outputs (29, 30). In Vietnam, women may believe that the husband is the family pillar, or support and that the wife's “heavenly” granted task is to take care of children and family members (19). Women are more responsible for food security, including raising some pigs or chickens, and men are more responsible for income generation (19, 31). Similarly, in Nepal, women spend more time caring for pigs than men (32); this is also the case in Tanzania, Ethiopia, and Nicaragua, where pig raising is considered to contribute to food security (33). In Vietnam, traditional task allocation may lead to inefficient use of resources which could be overcome by mass communication on gender equality, and/or by gender equality education for boys and girls (27).

The importance of women in veterinary input shops was not previously documented and implies gendered capacity-building should be incorporated in sales-based surveillance systems. Likewise, the higher risk of pig disease in women-dominated farms, implies that women should receive more training in veterinary drug use and pig disease management.

Compared with other countries in the Southeast Asia region, Vietnamese women have high decision-making powers (21, 27, 31). We found similar access to income generated from pig rearing for men and women, however, in Uganda, pig ownership and labor investment by women did not guarantee them decision-making power or benefits from pig-enterprise income (22). Even in Vietnam, most agreed that men tend to have the final say in important matters relating to land, housing, and purchasing valuable assets (19).

Our findings on the handling of dead pigs showed that females participants used better on-farm practices, either to prevent pig diseases or to ensure food safety, than male counterparts. Female pork retailers also were more positive the role of meat inspection. Greater concern by women over food safety and pig disease prevention were also reported in a recent study by Loan et al. (34), which would suggest tailoring interventions toward pig production practices, food safety certification, and tests according to gender groups would be beneficial.

While women contribute significantly in almost all nodes of the pig VCs, they are still burdened with household work (27). Additionally, the gendered divisions of labor in pig production expose men and women to different foodborne pathogens and occupational health risks (1), and misperception by pig VC actors on the risk of zoonotic disease was also observed by Sinh et al. (35). For example, a high *Salmonella* prevalence is reported (36, 37) and women are exposed to risk when clearing of offal and cleaning floors after slaughtering (38, 39) and as retailers, processors and consumers (37).

There have some limitations in the present study. First, the study was conducted in 2013-4, and smallholder pig VCs has tended to change due to the development, particularly because of the incursion of African swine fever. However, our findings are still representative of smallholder pig VCs, especially in the rural areas and moreover serve as a historical snapshot against which change can be evaluated. Second, dataset hierarchy by geography has not been factored in the analysis, which might affect statistical results and inference. Lastly, reliance on self-reporting may lead to bias.

## Conclusions

Women contribute significantly to and play major roles in smallholder pig VCs in Vietnam. They dominate in most of the chain nodes (e.g., production, processing, and retailing), especially in smaller pig farms and pork retailing. The gendered division of work varies with the scale of labor, with higher participation by men in larger-scale production. Women were mainly in charge of routine husbandry activities, while men were more often responsible for tasks requiring strength, knowledge, and skills. No significant difference in the financial performance of pig production was found between male- and female-dominated farms. Other important gendered issues pertaining to intra-household income include the ownership and use of said income, along with leisure time for both women and men within the family, and this was not investigated for all chain nodes. We recommend that future research on gendered roles in pig VCs study these aspects to confirm whether there are relevant gendered issues within the chain. Occupational risks for pig VC actors should also be studied, including gender aspects, and communicated to chain actors.

## Data availability statement

The data that support the findings of this study are available from the corresponding author, Sinh Dang-Xuan, upon reasonable request. Requests to access the datasets should be directed to, SD-X, s.dang@cgiar.org.

## Ethics statement

The studies involving human participants were reviewed and approved by the Institutional Review Board of the Hanoi School of Public Health, Hanoi, Vietnam. The patients/participants provided their written informed consent to participate in this study.

## Author contributions

DG, FU, HN-V, and NN-T-D conceptualized and designed the study. HD-N, HN-T-T, and TN-X conducted the field work and translated the raw data. HP-V, HN-T-D, TN-X, and HD-N performed the data processing and validation. NN-T-D, SD-X, HD-N, and HP-V analyzed the data. NN-T-D and SD-X prepared the draft manuscript. DG, HN-V, FU, NN-T-D, and SD-X reviewed and edited the manuscript. All authors have read and approved the final manuscript. All authors contributed to the article and approved the submitted version.

## Funding

This study was a part of the PigRISK project (Grant number: LPS/2010/047) and SafePORK project (Grant number: LPS/2016/143) funded by the Australian Center for International Agricultural Research (ACIAR), and the Consultative Group on International Agricultural Research (CGIAR), Research Program Agriculture for Nutrition and Health (A4NH).

## Conflict of interest

The authors declare that the research was conducted in the absence of any commercial or financial relationships that could be construed as a potential conflict of interest.

## Publisher's note

All claims expressed in this article are solely those of the authors and do not necessarily represent those of their affiliated organizations, or those of the publisher, the editors and the reviewers. Any product that may be evaluated in this article, or claim that may be made by its manufacturer, is not guaranteed or endorsed by the publisher.
