# Supplementary material for: Gender-focused analysis and opportunities for upgrading within Vietnam's smallholder pig value chains
Source: Front Vet Sci. 2022 Aug 9;9:906915. doi: 10.3389/fvets.2022.906915 (PMC9395733; doi:10.3389/fvets.2022.906915)
Supplement: Supplementary file 1 [file Table_1.docx]

Supplementary Material

# Supplementary Figures and Tables

## Supplementary Tables

**Supplementary Table 1.** Disease risk and management in pig production by males and females.

| **Parameters** | **Male**  (n=204) | **Female**  (n=216) | **Overall** | ***p*-value^†^** |
| --- | --- | --- | --- | --- |
| Sick piglets were accidentally bought last year (%) | 5.8 | 11.4 | 9.3 | 0.037^a^ |
| Sick pigs were present during raising at farm last year (%) |  |  |  |  |
| Piglets | 32.2 | 23.6 | 27.0 | 0.046^a^ |
| Growing pigs | 5.7 | 4.4 | 4.9 | 0.721^a^ |
| Pigs died during raising at farm last year (number of pigs) | 1.9 | 1.8 | 1.8 | 0.488^b^ |
| Monetary loss due to sick and dead pigs last year (US$) | 83.5 | 97.4 | 91.8 | 0.117^b^ |

Note: (**†**) *p*-values were derived from comparison between male and female groups, (^a^) using Chi square tests, (^b^) using *t-*tests.
