# Supplementary material for: Gender-focused analysis and opportunities for upgrading within Vietnam's smallholder pig value chains
Source: Front Vet Sci. 2022 Aug 9;9:906915. doi: 10.3389/fvets.2022.906915 (PMC9395733; doi:10.3389/fvets.2022.906915)
Supplement: Supplementary file 2 [file Image_1.pdf]

Supplementary Material

1 Supplementary Figures and Tables

1.1 Supplementary Figures

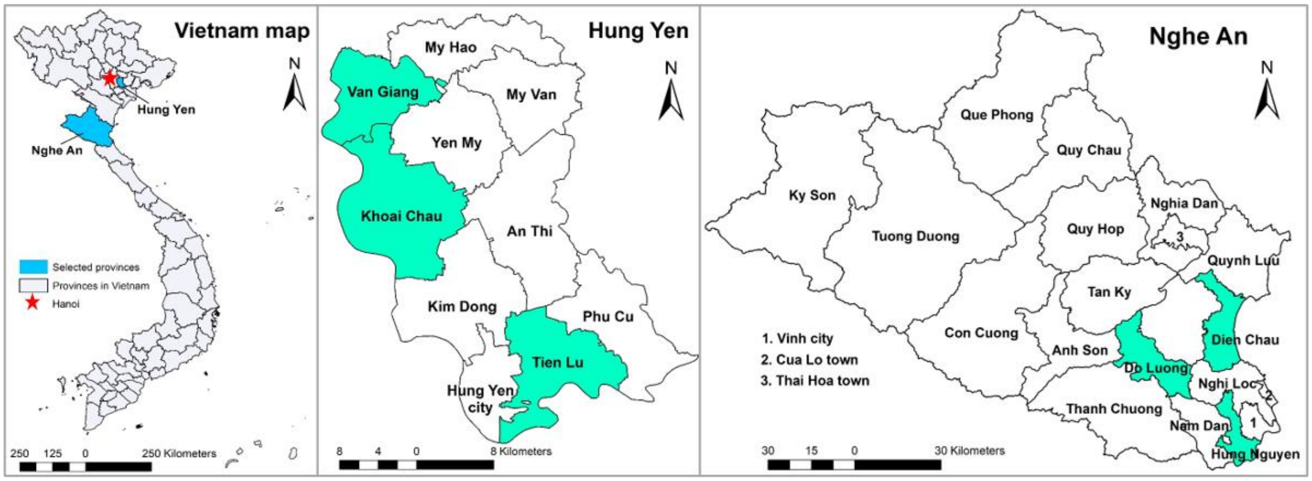

Supplementary Figure 1. Hung Yen and Nghe An provinces and selected districts in the study.
Left: map of Vietnam, with selected provinces highlighted blue.
Middle and Right: maps of the Hung Yen and Nghe An provinces, with the selected districts highlighted green.
