# Supplementary material for: Gender-focused analysis and opportunities for upgrading within Vietnam's smallholder pig value chains
Source: Front Vet Sci. 2022 Aug 9;9:906915. doi: 10.3389/fvets.2022.906915 (PMC9395733; doi:10.3389/fvets.2022.906915)
Supplement: Supplementary file 3 [file Image_2.pdf]

Supplementary Material

1 Supplementary Figures and Tables

1.1 Supplementary Figures

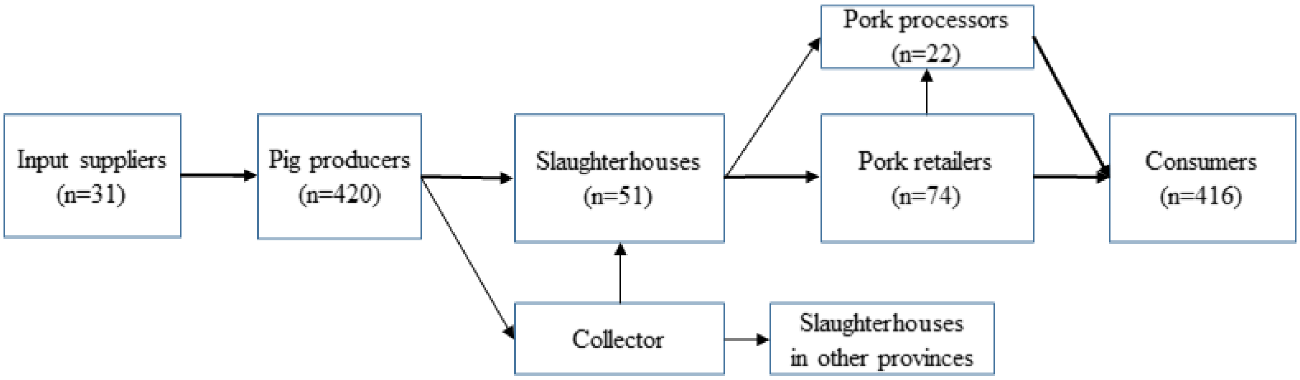

Supplementary Figure 2. Schematic of smallholder pig value chains in Vietnam.
Numbers in parentheses = number of respondents in the study corresponding to each group in the value chain.
Arrows represent the flow of pigs/pork products through the chain.
